# Memphis Dental Society

**Published:** 1867-12

**Authors:** W. T. Arrington, W. D. Tucker

**Affiliations:** Memphis, Tenn.; Memphis, Tenn.


### Proceedings of Societies.

### MEMPHIS DENTAL SOCIETY.

MEMPHIS, Tenn., Oct. 15th, 1867.

SIR :—At a meeting of the Memphis Dental Society, held at the rooms of Dr. W. T. Arrington, on the first Tuesday in September last, and the first anniversary of the society, the following gentlemen were elected to fill the various offices for the ensuing year, viz.:

*President*—Dr. W. T. Arrington.
*Vice-President*—Dr. V. F. Elliott.
*Corresponding Secretary*—S. Hinson.
*Recording Secretary*—Dr. W. D. Tucker.
*Treasurer*—Dr. G. W. Acree.
*Librarian*—Dr. S. A. Smith.

The following gentlemen were appointed an Executive Committee for one year:

Drs. J. B. Wasson, J. C. Harris, and E. W. Sawyer.

At a meeting held on the 17th September, a Committee was appointed to investigate and report upon the status of the St. Louis Dental College, and on the 2d of October the following report was rendered, and received the unanimous sanction of the society.

We the Committee appointed to investigate and report upon the status of the St. Louis Dental College, beg leave to submit the following as our report:

Having made a thorough investigation of the causes that led to its establishment, and the present status of the St. Louis Dental College, feel that it cannot be endorsed by us as a society of Dentists, from the fact that its faculty was not, prior to its organization, regular Doctors of Dental

Surgery. They are a self-constituted and self-graduated faculty, more than one of whom applied to and was rejected by the examining board of the Missouri Dental College. Therefore,—

*Resolved*, That we the Memphis Dental Society, ignore all Dental Colleges which are not represented in the American Dental Association, and would earnestly recommend to all students of Dentistry, who have any aspirations to complete a thorough and regular course of scientific studies, to make diligent inquiries before selecting and entering a Dental college; further,—

*Resolved*, That we do most heartily endorse the action of the American Dental Association, held at Cincinnati in July last, in reference to this the St. Louis Dental College.

W. T. Arrington, *President.*

W. D. Tucker, *Secretary.*

